# Advanced Materials Structures for Sound and Vibration Damping

**DOI:** 10.3390/ma15041295

**Published:** 2022-02-10

**Authors:** Martin Vašina

**Affiliations:** Department of Physics and Materials Engineering, Tomas Bata University in Zlín, Vavrečkova 5669, 760 01 Zlín, Czech Republic; vasina@utb.cz

The studies of sound and vibration are closely related. Noise is an unwanted sound that is considered unpleasant, loud, or disturbing to the organs of hearing. Mechanical vibration is caused by the oscillation of a mechanical or structural system around its equilibrium position. Noise and mechanical vibration are, in many cases, among the negative environmental factors. They can have an adverse effect on human health, production accuracy, the life of processing equipment and tools, labor protection, etc. For these reasons, it is necessary to eliminate unwanted noise and mechanical vibration by appropriate means. There are various options to reduce excessive noise and mechanical vibration. In general, it is necessary to convert the excessive mechanical energy of oscillating motion and acoustic energy into other types of energy, especially heat.

This Special Issue focuses on the collection of scientific papers dealing with the development of advanced material structures for sound and vibration damping. Various measures that can help eliminate unwanted noise and mechanical vibration are explored in this Special Issue.

In [1], the authors investigated the mechanical, acoustic, and thermal properties of cellular rubber composites, which were produced with different concentrations of silica nanofillers at the same blowing agent concentration. It was found that a higher concentration of silica nanofillers generally led to an increase in the mechanical stiffness and thermal conductivity and to a decrease in the sound absorption and thermal degradation of the investigated rubber composites. It was also possible to find a correlation between the mechanical stiffness of the tested rubber composites evaluated using conventional and vibroacoustic measurement techniques. Vibration damping and sound absorption methods are relatively simple, inexpensive, fast, and non-destructive compared to the conventional methods used to determine the mechanical stiffness of solids. Therefore, the vibroacoustic methods can also be easily applied to compare the mechanical stiffness of different materials.

Monkova et al. [2] studied the sound absorption properties of 3D printed open porous acrylonitrile butadiene styrene (ABS) specimens that were produced with four different lattice structures (Cartesian, Starlit, Rhomboid, and Octagonal). In this work, various factors affecting the sound absorption properties were evaluated, namely, the type of lattice structure, the excitation frequency of acoustic waves, the specimen thickness, and the air gap size behind the sound-absorbing materials inside the acoustic impedance tube. It could be concluded that the application of 3D printed materials is promising in terms of sound absorption. Three-dimensional printing technology allows the production of lightweight materials of various shapes and structures compared to other manufacturing technologies, which saves time and energy and reduces the weight of materials.

Muhazeli et al. [3] dealt with the sound-damping properties of a magneto-induced (called magnetorheological) foam containing different concentrations of carbonyl iron particles. It was found that the addition of a magnetic field led to a peak frequency shift from the middle to higher frequency ranges. Therefore, the change in the magnetic field has a significant influence on sound absorption, and the MR foam could be applied as a noise-controllable material.

The vibration-damping properties of environmentally friendly concrete using crumb rubber recycled from waste rubber tires were investigated in [4]. It could be concluded that a mix of 180- and 400-micron crumb rubber significantly improved the concrete’s damping ratio (improvement of 100%) compared to normal concrete. For this reason, the use of crumb rubber in concrete presents one of the best alternatives while dealing with rubber waste, as it will help both environmental protection and the reduction in railway sleeper costs.

The effect of conditioning on the vibration damping of polyurethane (PU) foams, which were subjected to conditioning at two different temperatures (45 and 80 °C) and relative humidity values (45 and 80%) for different time intervals, was evaluated in [5]. It was found that the conditioning process had a significant influence on the properties of the tested PU foams. In general, the thermal degradation and permanent deformation of the PU foams increased with the increasing conditioning time and temperature. The higher permanent deformation was accompanied by a decrease in Young’s modulus of elasticity and subsequently confirmed by non-destructive dynamical-mechanical vibration tests, which confirmed the samples’ higher vibration damping, resulting in the loss of elasticity.

The author of [6] investigated the optimization of the natural frequencies of circular cylindrical shells using axially functionally graded materials. The constituents of the functionally graded materials (FGMs) were graded in the axial direction. It could be concluded that the spatial change in material properties changed the structural stiffness and mass, which then affected the structure’s natural frequencies. Therefore, axially FGMs can be a useful technique for the optimization of natural frequencies.

Martin Vašina is an academic and researcher at Tomas Bata University in Zlín and at VŠB-Technical University of Ostrava in the Czech Republic. He deals mainly with mechanical, vibration isolation, sound absorption and light-technical properties of various materials. Another part of his research is focused on fluid mechanics. He is a member of the Czech Society for Mechanics.
